# Systematic analysis of CCCH zinc finger family in *Brassica napus* showed that *BnRR-TZF*s are involved in stress resistance

**DOI:** 10.1186/s12870-021-03340-8

**Published:** 2021-11-23

**Authors:** Boyi Pi, Jiao Pan, Mu Xiao, Xinchang Hu, Lei Zhang, Min Chen, Boyu Liu, Ying Ruan, Yong Huang

**Affiliations:** 1 College of Bioscience and Biotechnology, Hunan Agricultural University, Changsha, 410128 China; 2 Key Laboratory of Crop Epigenetic Regulation and Development in Hunan Province, Changsha, 410128 China; 3 College of Agronomy, Hunan Agricultural University, Changsha, 410128 China

**Keywords:** CCCH-type transcription factor, Tandem CCCH zinc finger, Evolution, Abiotic stress, *Brassica napus*

## Abstract

**Background:**

CCCH zinc finger family is one of the largest transcription factor families related to multiple biotic and abiotic stresses. *Brassica napus* L., an allotetraploid oilseed crop formed by natural hybridization between two diploid progenitors, *Brassica rapa* and *Brassica oleracea*. A systematic identification of rapeseed CCCH family genes is missing and their functional characterization is still in infancy.

**Results:**

In this study, 155 *CCCH* genes, 81 from its parent *B. rapa* and 74 from *B. oleracea*, were identified and divided into 15 subfamilies in *B. napus*. Organization and syntenic analysis explained the distribution and collinearity relationship of *CCCH* genes, the selection pressure and evolution of duplication gene pairs in *B. napus* genome. 44 diploid duplication gene pairs and 4 triple duplication gene groups were found in *B. napus* of CCCH family and the segmental duplication is attributed to most *CCCH* gene duplication events in *B. napus*. Nine types of CCCH motifs exist in *B. napus* CCCH family members, and motif C-X_7/8_-C-X_5_-C-X_3_-H is the most common and a new conserved CCH motif (C-X_5_-C-X_3_-H) has been identified. In addition, abundant stress-related cis-elements exist in promoters of 27 subfamily IX (*RR-TZF*) genes and their expression profiles indicated that *RR-TZF* genes could be involved in responses to hormone and abiotic stress.

**Conclusions:**

The results provided a foundation to understand the basic characterization and genes evolution of *CCCH* gene family in *B. napus*, and provided potential targets for genetic engineering in Brassicaceae crops in pursuit of stress-tolerant traits.

**Supplementary Information:**

The online version contains supplementary material available at 10.1186/s12870-021-03340-8.

## Introduction

Plant transcription factors (TFs) play an important role in the regulation of plant growth, development, and environmental stress responses. A large number of transcriptional factors function in abiotic stress, such as drought, saline-alkali, extreme temperature and other stresses. Transcription factors bZIP [1, 2], WRKY [3], AP2/ERF [4], MYB [5] bHLH [6], NAC [7], GRAS [8], HD-ZIP [9] and ZFP [10] are known more in response to abiotic stresses. Zinc Finger Protein (ZFP) family is one of the largest transcription factor families, which contains nine different types, including C2H2, C3H, C3HC4, C2HC5, C4HC3, C2HC, C4, C6, and C8 according to the number of their conserved Cys (C) and His (H) motifs [11]. Among them, the CCCH-type transcription factors, one of the most widespread ZFPs in eukaryotes, consist of three cysteines and a histidine coordinated by zinc ion [12].

The typical CCCH Zinc Finger proteins always harbor 1–6 CCCH repeated motifs, and C-X_7/8_-C-X_5_-C-X_3_-H is the most ubiquitous motif. CCCH Zinc Finger proteins are divided into two types: the Tandem CCCH-type Zinc Finger (TZF) and the non-TZF proteins based on the number and distribution of CCCH motifs. TZF proteins only contain two tandem CCCH-type zinc finger motifs whereas non-TZF proteins have one or more than two CCCH-type zinc finger motifs [13]. Both the non-TZF and the TZF genes play important roles in many biological processes including the development process, biotic and abiotic stresses [13]. For example, non-TZF gene *AtC3H17*, as a nuclear transcriptional activator, promoted seed germination, seedling development and caused early-flowering through activated transcription of *OLEO1*, *OLEO2* and *CRU3* by binding onto their promoters in Arabidopsis [14] and enhanced the resistance of osmotic, oxidative and salt stresses by positively regulating the ABA-dependent stress-response pathway [13]. *IbC3H18* could be induced by NaCl, polyethylene glycol (PEG), H_2_O_2_ and abscisic acid (ABA) and interact with *IbPR5* to enhance salt and drought tolerance [15]. Plant TZF proteins are evolutionarily conserved regulators in growth and responses to hormones and stresses [16]. *OsC3H10*, a TZF gene in rice, was demonstrated to participate in the regulation of the drought tolerance pathway by elevating the expression of stress-related genes [17]. A plant-unique arginine-rich (RR) region located in the front of C-X_7/8_-C-X_5_-C-X_3_-H- X_16_-C-X_5_-C-X_4_-C-X_3_-H (TZF) motif [18]. Both the RR and TZF domains are essential to RNA binding in Arabidopsis [18]. RR-TZF proteins subfamily is one of the largest CCCH subfamilies in plant species, 11 of 68 in Arabidopsis [19], 16 of 91 in poplar [20], 17 of 103 in *Brassica rapa* [21], 16 of 103 in switchgrass [22], 25 of 89 in banana [23] and 12 of 119 in *Moso Bamboo* [24]. Plant RR-TZF proteins can further be divided into two groups: the RR-TZF containing RR and TZF domain, and the ANK-RR-TZF containing an extra ANK (Ankyrin) domain. Arabidopsis RR-TZF members *AtTZF1, AtTZF2, AtTZF3, AtTZF4, AtTZF5* and *AtTZF6 (AtTZF1–6)* belong to ANK-RR-TZF and their functions are more diversified. *AtTZF1–3* functions in ABA-mediated drought tolerance and JA-induced senescence while *AtTZF4–6* are negative regulators of seed germination [25]. *AtTZF7, AtTZF9, AtTZF10, AtTZF11* could positively regulate vegetative growth and be involved in abiotic stress tolerance responses [16]. Ankyrin (ANK) is ubiquitous in eukaryotes, prokaryotes and viruses and ANK family members are involved in light signal regulation, embryonic development, leaf morphogenesis, lateral root formation and so on [26].

CCCH zinc finger proteins might be involved in organism development and stress response through post-transcriptional regulation. Human ZFP36 (Tristetraprolin, TTP) is the prototype of the mammalian TZF that consists of two tandem CCCH motifs inserted with 18 amino acids [18]. TTP or Arabidopsis TZF1 promotes the degradation of mRNA by inhibiting the assembly of target mRNA polyA through combining to a specific site of the 3′-UTR region (UUAUUUAUU) of target genes [18, 27]. Likewise, ZFP36L2 is another TZF protein in animal kingdom. And it was known as a very unstable mRNA binding protein that controls maternal fertility and physiological function during early embryonic development [28, 29].

*Brassica napus*, one of the most important oil crops of Brassicaceae, is an allotetraploid hybrid of *Brassica rapa* (A-subgenome, AA, *n* = 10) and *Brassica oleracea* (C-subgenome, CC, *n* = 9) [30]. The function of *CCCH* genes in *B. napus* is little known except that overexpression of CCCH-type transcription factor *BnZFP1* increased in oleic acid and oil levels in *B. napus* by positive regulation of its target gene diacylglycerol O-acyltransferase 1 (*DGAT1*) [15]. In fact, the roles of rapeseed *CCCH* genes in development and abiotic stress are known much little. In this study, *CCCH* genes of *B. napus* on whole genome level were identified and their expression in response to abiotic stresses were investigated, and these results provided a foundation for further research in *CCCH* genes.

## Results

### Identification and chromosome localization of *CCCH* genes in *B. napus*

**Fig. 1:**
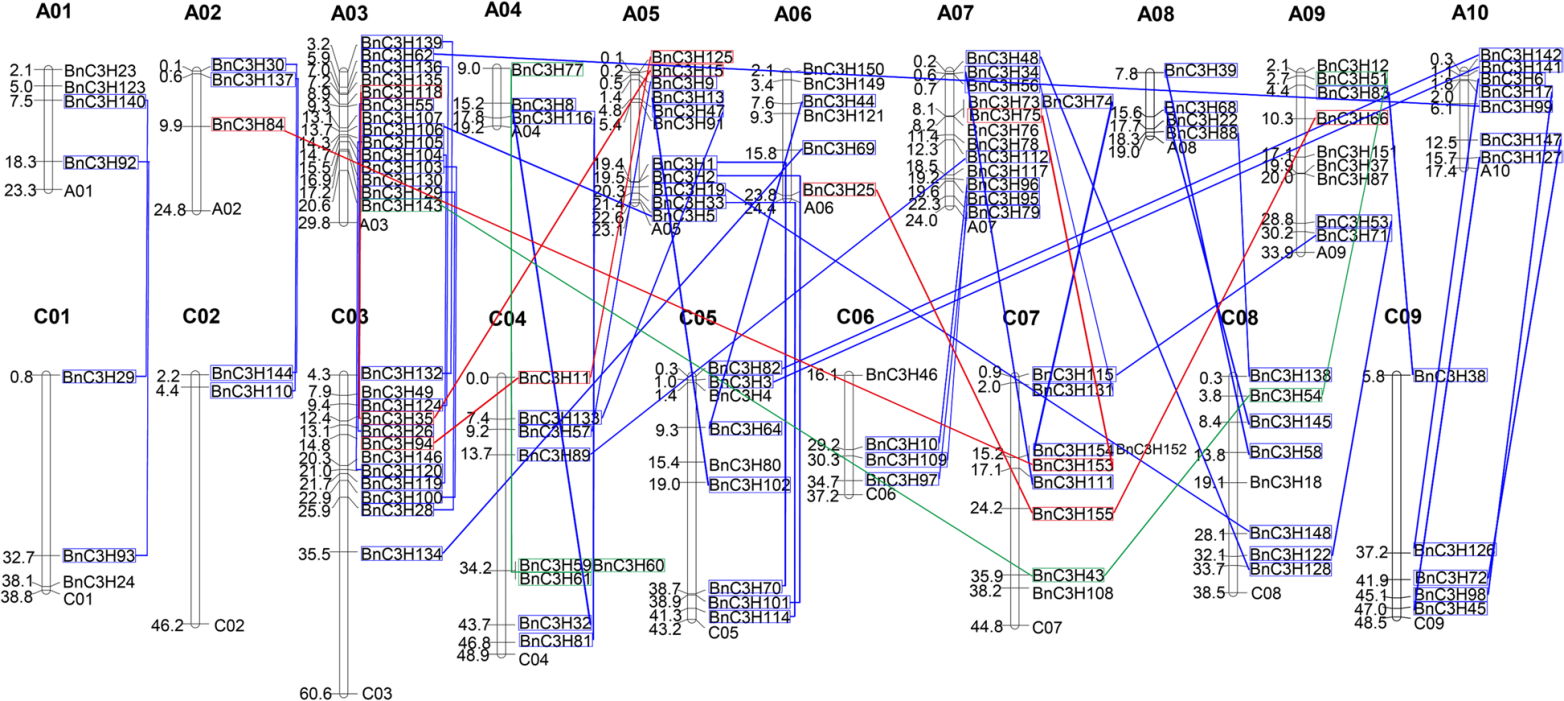
Chromosomal locations of *CCCH* genes in *B. napus*. The 135 of 155 *CCCH* genes are located on chromosome A01-A10 and chromosome C01-C09. The scale on the chromosome represents megabases (Mb) and the chromosome number is indicated at the top of each chromosome

**Fig. 2:**
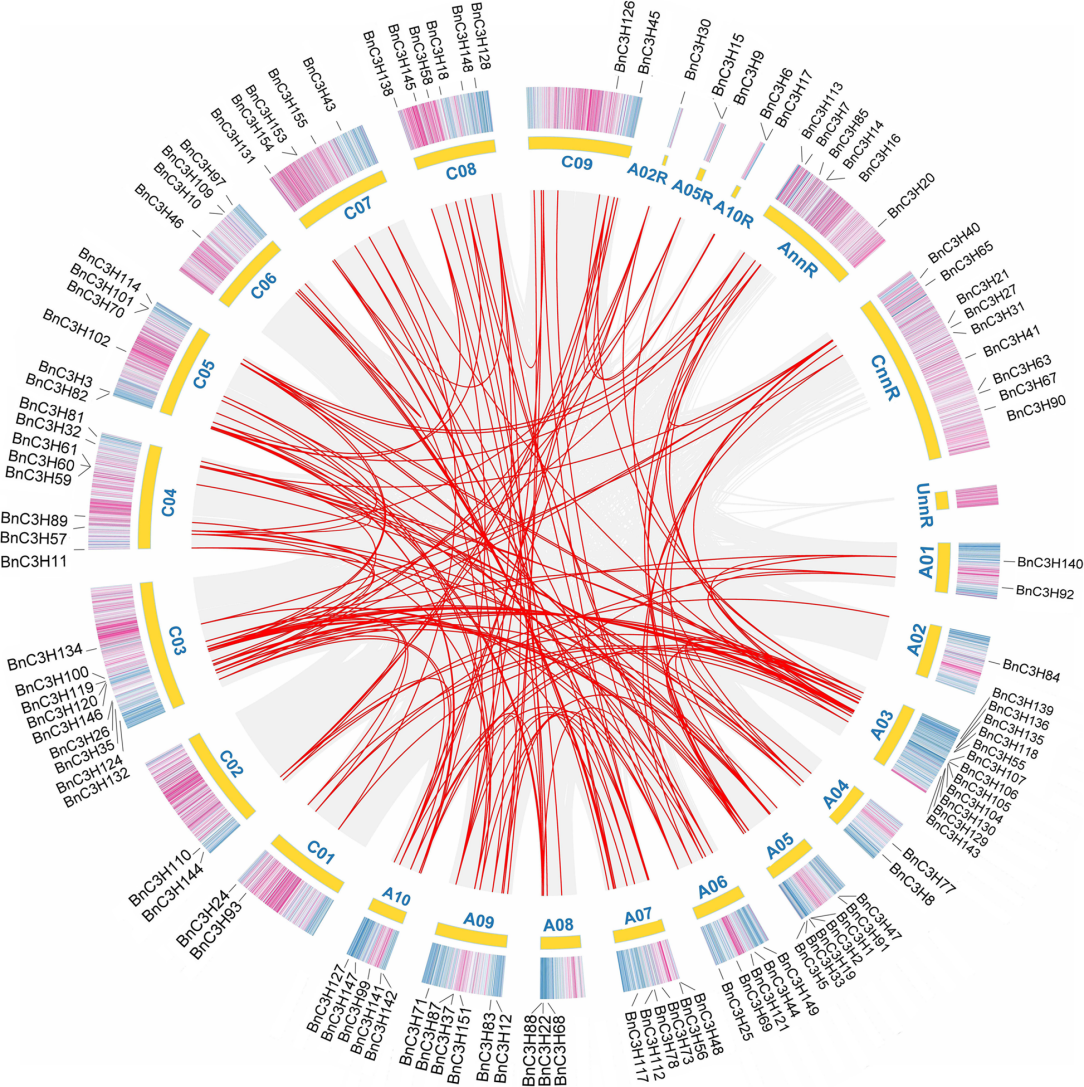
Distribution on chromosomes and colinear analysis of *CCCH* gene in *B. napus*. The distribution map was drawn about the whole gene position, gene length, chromosome size, and detailed position of *CCCH* genes. The gray lines indicate collinear blocks in the whole *B. napus* genome, and the red lines colinear gene pairs in CCCH family in *B. napus*

One hundred and fifty-five *CCCH* genes were identified by Blastp tools in *B. napus* database. The subgenome A possesses more *CCCH* genes than the subgenome C. 81 *CCCH* genes evolve from the subgenome A and 74 *CCCH* genes evolve from the subgenome C (Additional file 1). The 135 of 155 *CCCH* genes are located in ChrA01-A10, ChrC01-C09 (Figs. 1 and 2). Besides, the chromosome localization of the other 20 *CCCH* genes in *B.napus* is unknown. Among A-subgenome, 74 *CCCH* genes locate in ChrA1-A10 chromosome while 7 *CCCH* genes are unconfirmed. ChrA09 (33.9 M), the longest chromosome in the A genome, carries 9 *CCCH* genes. And ChrA03 (29.8 M) carries the largest number of 14 *CCCH* genes. Second to ChrA03, ChrA07 (24.0 M) contains 13 *CCCH* genes. Among C-subgenome, 61 *CCCH* genes locate in Chr1-Chr9, whereas 13 *CCCH* genes are still on the scaffold. ChrC03 (60.6 M), the longest chromosome in A and C, has 12 *CCCH* genes.

### Gene collinearity and duplication of *CCCH* in *B. napus*

Most *CCCH* orthologous genes in *B. rapa* and *B. oleracea* remain as homeologous gene pairs in *B. napus*. There are 24 collinearity *CCCH* gene pairs only in subgenome A, 16 collinearity *CCCH* gene pairs only in subgenome C, and 92 collinearity *CCCH* gene pairs between subgenome A and subgenome C in *B. napus* (Fig. 2, Additional file 2). Comparative analysis with the parent genomes revealed that the *B. napus* genome retained 98.6% *CCCH* genes of *B. oleracea* (74 of 75) in comparison to only 78.6% *CCCH* genes of *B. rapa* (81 of 103) (Additional file 1).

The organism gene duplication occurred through segmental, or tandem or whole genome [31]. Tandem and segmental duplication occurred when two or three closely related *BnC3H* genes were located on the same or different chromosomes [32]. Reference on the collinearity of the *CCCH* family (Fig. 2, Additional file 2) and the criteria of Yang [33], the duplication events have occurred among 108 genes which were disseminated in 10 A-chromosomes and 9 C-chromosomes (Additional file 3). Among them, forty-four diploid duplication gene pairs, four triploid duplication gene groups and two quadruple duplication gene groups were found (Additional file 3). The results showed that most *CCCH* duplication gene pairs are segmental duplication except three tandem duplications pairs, *BnC3H59/BnC3H60, BnC3H60/BnC3H61 and BnC3H59/BnC3H61* in *B. napus CCCH* family (Additional file 3). There are eight gene pairs have been identified between ChrA03 and ChrC03, which have the highest frequency diploid duplication. The most diploid duplication and quadruple duplication genes groups occurred between ChrA05 and ChrC05, ChrA04 and ChrC04, respectively (Additional file 3, Fig. 1). It might suggest that duplication events also happened between A- and C- subgenomes in process of *B. napus* formation.

The selection mode of the coding sequences can be predicted through the Ka/Ks ratio. In *B. napus*, the Ka/Ks ratio of segmentally duplicated *CCCH* gene pairs were < 1 (the majority Ka/Ks ratio < 0.5), and it suggested that duplicated *BnC3H* gene pairs were under purifying negative selection. Additionally, the duplication events might occur less than 10 MYA in *B. napus* (Additional file 3).

### Phylogenetic relationship analysis of CCCH family

**Fig. 3:**
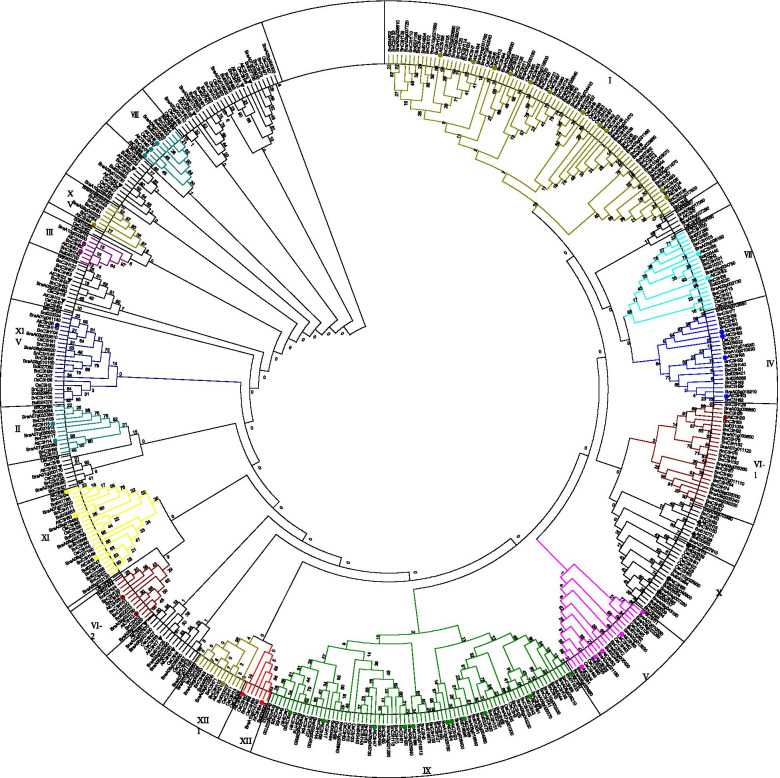
Phylogenetic tree of CCCH proteins in *B. napus*. The phylogenetic tree contained CCCH proteins of 155 *B. napus*, 68 *A. thaliana,* 67 *O. sativa,* 102 *B. rapa and* 75 *B. oleracea*. 134 CCCH proteins were divided into 15 subfamilies and 21 CCCH proteins were not grouped in *B. napus.* All Arabidopsis CCCH proteins are specially labeled and each line color and the letters in the ring represent a different branch. Protein sequences were aligned using MUSCLE and the tree was generated by MEGA 7 (Statistical Method: Maximum Likelihood; Model/Method: Poisson model; Gaps/Missing Data Treatment: Use all sites; No. of Bootstrap Replications: 1000; Branch Swap Filter: Weak)

To further explore the diversity and conservation of BnC3H proteins, the 155 CCCH full-length protein sequences were used to construct a phylogenetic tree by the Maximum Likelihood (ML) method (Fig. 3). 134 CCCH proteins were divided into 15 subfamilies, and 21 CCCH proteins were not confirmed. Subfamily I is the largest clade with 34 CCCH proteins, followed by the subfamily IX RR-TZF with 27 CCCH proteins. Besides, the subfamily V and subfamily X only has one CCCH member. Compared with Arabidopsis and rice, the subfamily VI is pretty special in that the three BnC3H proteins are divided into two groups.

### Gene structure and protein structure of CCCH zinc finger in *B. napus*

**Fig. 4:**
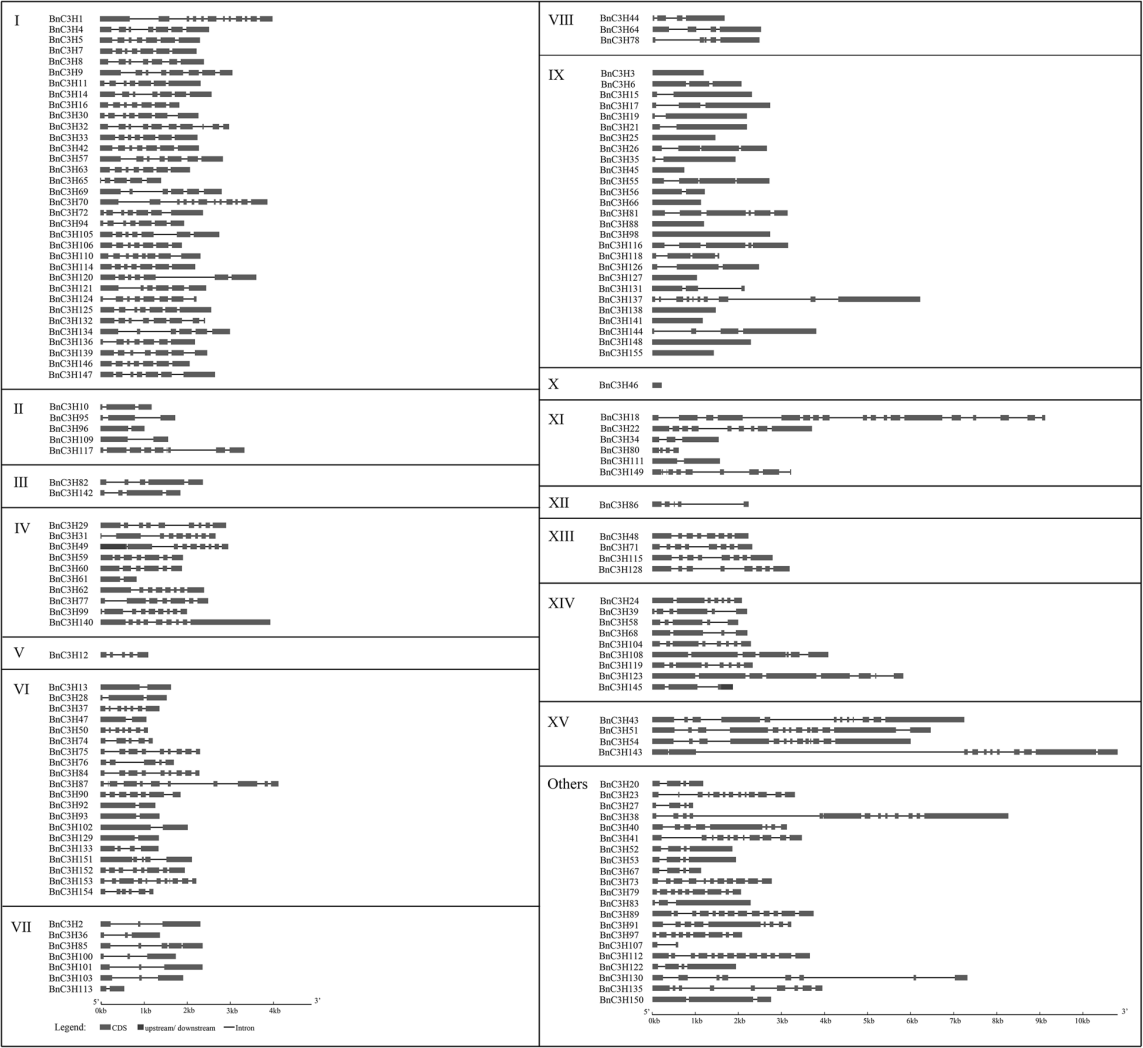
The gene structure of *CCCH* genes in *B. napus*. The exons and introns of *BnC3H* genes varied from 1 to 18, but the gene structure of *C3H* in each family was relatively conservative except subfamily XI. The gene structure of 155 *CCCH* genes was constructed by Gene Structure Display Server (http://gsds.gao-lab.org/index.php)

To pinpole the evolution trajectory and study the function diversity of *B. napus CCCH* genes, the gene structure of *BnC3Hs* was analyzed. It was found that the exons and introns of *BnC3H* genes varied from 1 to 18, but the gene structure of *CCCH* in each family was relatively conservative except subfamily XI genes structure diversify variety (Fig. 4). The number of exons of subfamily I is relatively conservative than others, ranging from 5 to 8. In terms of the structure of subfamily IX, they behaved in two types of the structure of genes. 11 of them only have one exon, no introns, and the rest of them all have 2–5 exons except *BnC3H137* and *BnC3H81*. This family can be classified into two groups. The longest genes were in subfamily XV and 4 members of them possess 11–13 exons. *BnC3H18* possesses the largest number of exons (18) in subfamily XI. (Fig. 4).

**Fig. 5:**
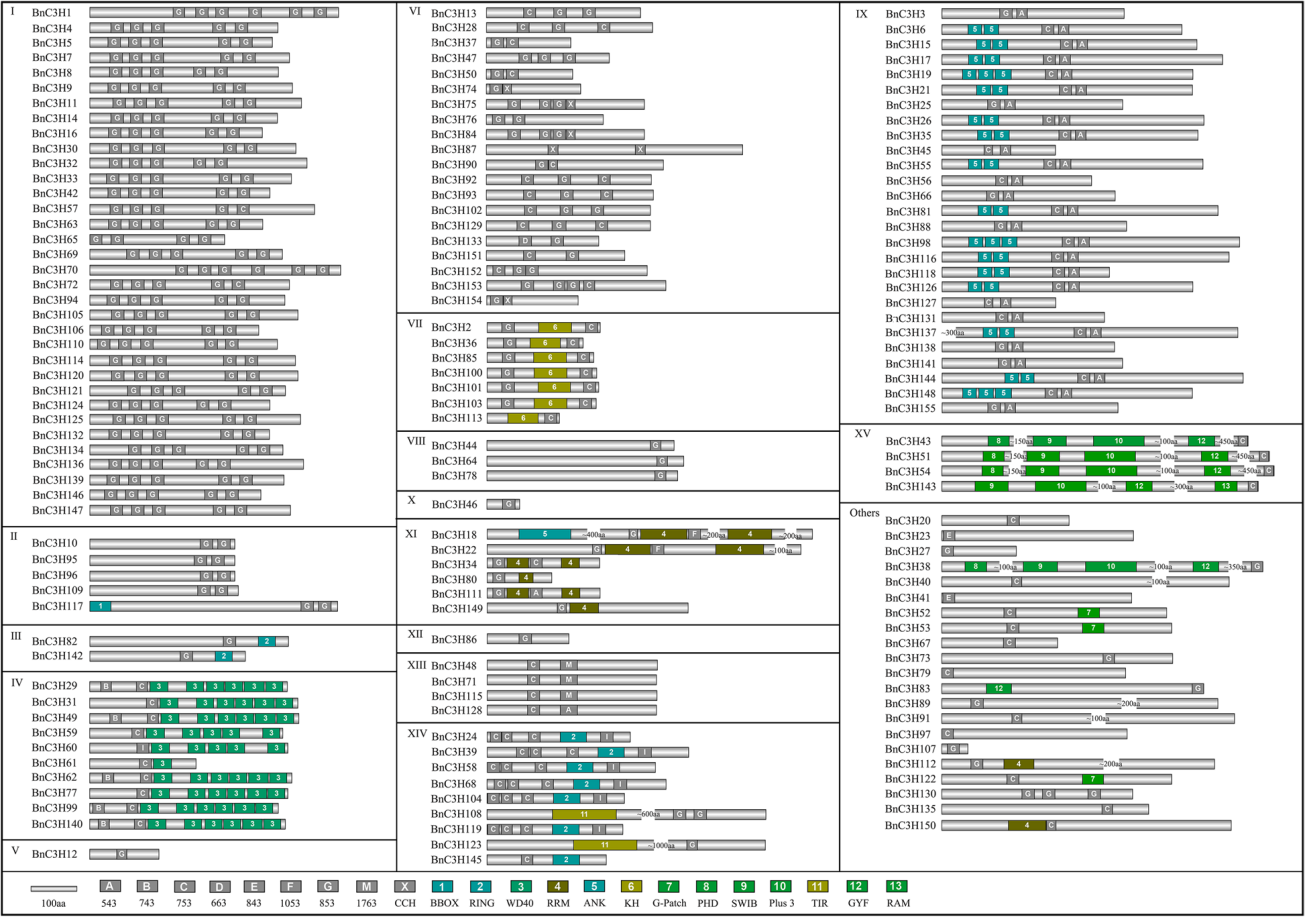
Conserved domain of CCCH proteins in *B. napus*. Nine different types of CCCH motifs were found in 155 BnC3H proteins. Each C3H protein contained 1–6 CCCH motifs in *B. napus*. The conserved domain of CCCH proteins was detected by SMART and NCBI (http://www.ncbi.nlm.nih.gov/Structure/cdd/wrpsb.cgi), and the Expect Value was set at 10. The site of domains information was constructed on IBS1.0.3 software

Domains are the building blocks of proteins. During evolution, domains produce novel structures and functions of proteins [34]. The results showed that there were great differences in the structure of CCCH  proteins. Nine different types of CCCH motifs were found in 155 CCCH proteins of *B. napus* (Fig. 5; Additional file 4). Each CCCH protein contained at least 1–6 CCCH motifs. The number and type of BnC3H proteins in each subgroup are relatively conservative. C-X_7–8_-C-X_5_-C-X_3_-H motif is the most common and extensive CCCH motif in *B. napus*, and it mainly occurred in the subfamily I, II, III, IV, VII, VIII and XV. Most proteins of subfamily I have five conserved C-X_7–8_-C-X_5_-C-X_3_-H motifs except BnC3H1 and BnC3H70 with six CCCH motifs. Subfamily IX CCCH proteins contain two conserved CCCH motifs divided by 18 amino acids (C-X_7–8_-C-X_5_-C-X_3_-H-X_16–18_-C-X_5_-C-X_4_-C-X_3_-H). The protein length of subfamily XV is the longest in *B. napus* CCCH family, and the four members are above 1000 amino acids length with a conserved C-X_7_-C-X_5_-C-X_3_-H motif. Interestingly, six special C-X_5_-C-X_3_-H motifs consisted of two cysteines (C) and one histidine (H) was found in *B. napus* subfamily VI. In addition to CCCH motifs, RING, WD40, KH, ANK and RRM domains also appeared conservatively in subfamily III, IV, VII, IX and XI (Fig. 5).

### Conserved structure of subfamily IX in *B. napus*

RR-TZF family plays an important role in plant growth, development and stress response [19]. To identify the *RR-TZF* family genes to respond to stress in *B. napus*, the promoter elements and RR-TZF domain composition of subfamily IX were analyzed.

**Fig. 6:**
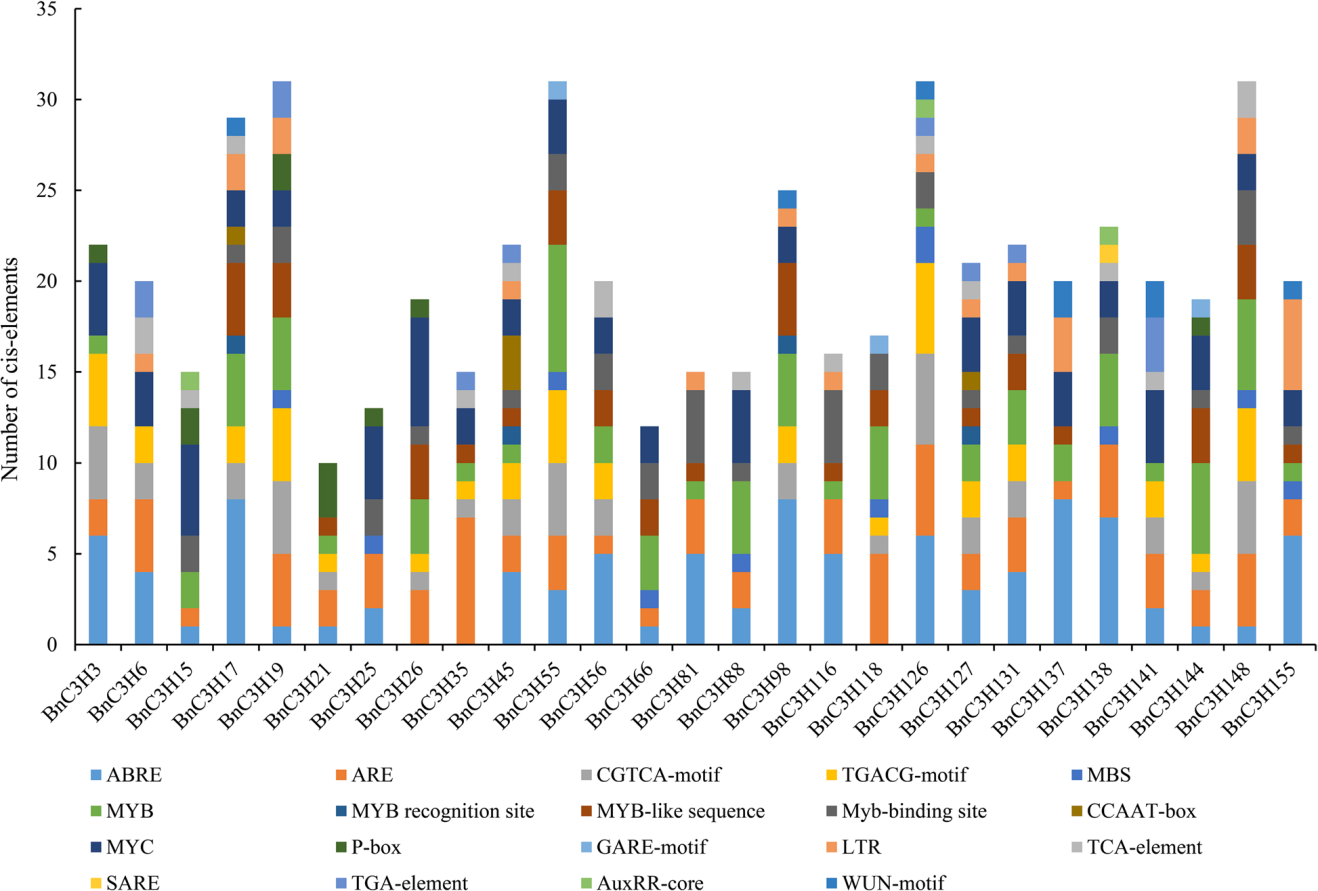
Type and number of cis-elements of *CCCH* genes of subfamily-IX in *B. napus*. A large number of cis-elements related to abiotic stress were widely found in BnRR-TZF subfamilies. The 1500 bp upstream region of *BnRR-TZF* genes is downloaded on a website (https://www.genoscope.cns.fr/brassicanapus/)

The promoter elements of the *RR-TZFs* were predicted on the PlantCARE (http://bioinformatics.psb.ugent.be/webtools/plantcare/html/). The results showed that all the *BnRR-TZF* promoters possessed typical CAAT and TATA boxes which are the core cis-acting element in promoter and enhancer regions. Except for the basic promoter elements, a large number of cis-elements related to abiotic stress were widely found. They could be grouped into three types, hormone-responsive elements, stress-responsive elements and light-responsive elements (Additional file 5, Fig. 6). For example, the ABA response element (ABRE) cis-elements related to the ABA response exist in almost all *RR-TZF* promoters except *BnC3H26*, *BnC3H35* and *BnC3H118*. Except for *BnC3H25*, *BnC3H66*, *BnC3H81*, *BnC3H88*, *BnC3H116*, *BnC3H137*, *BnC3H138* and *BnC3H155*, the promoters of other genes contain 1 to 5 elements: CGTCA-motif and TGACG-motif that related to jasmonic acid response, GARE-motif and P-box that associated with GA-induced plant growth regulation [35], while TGA-element and AuxRR-core that related to abiotic stress induced by a hormone, and MYB is a binding site of drought-induced genes, related to drought or drought stress caused by other abiotic stresses [36]. The results showed that a large number of promoter cis-elements of *BnRR-TZF* family genes were related to hormone and drought-induced abiotic stress (Fig. 6). Therefore, *BnRR-TZF* genes may be involved in hormone and drought-induced abiotic stress.

**Fig. 7:**
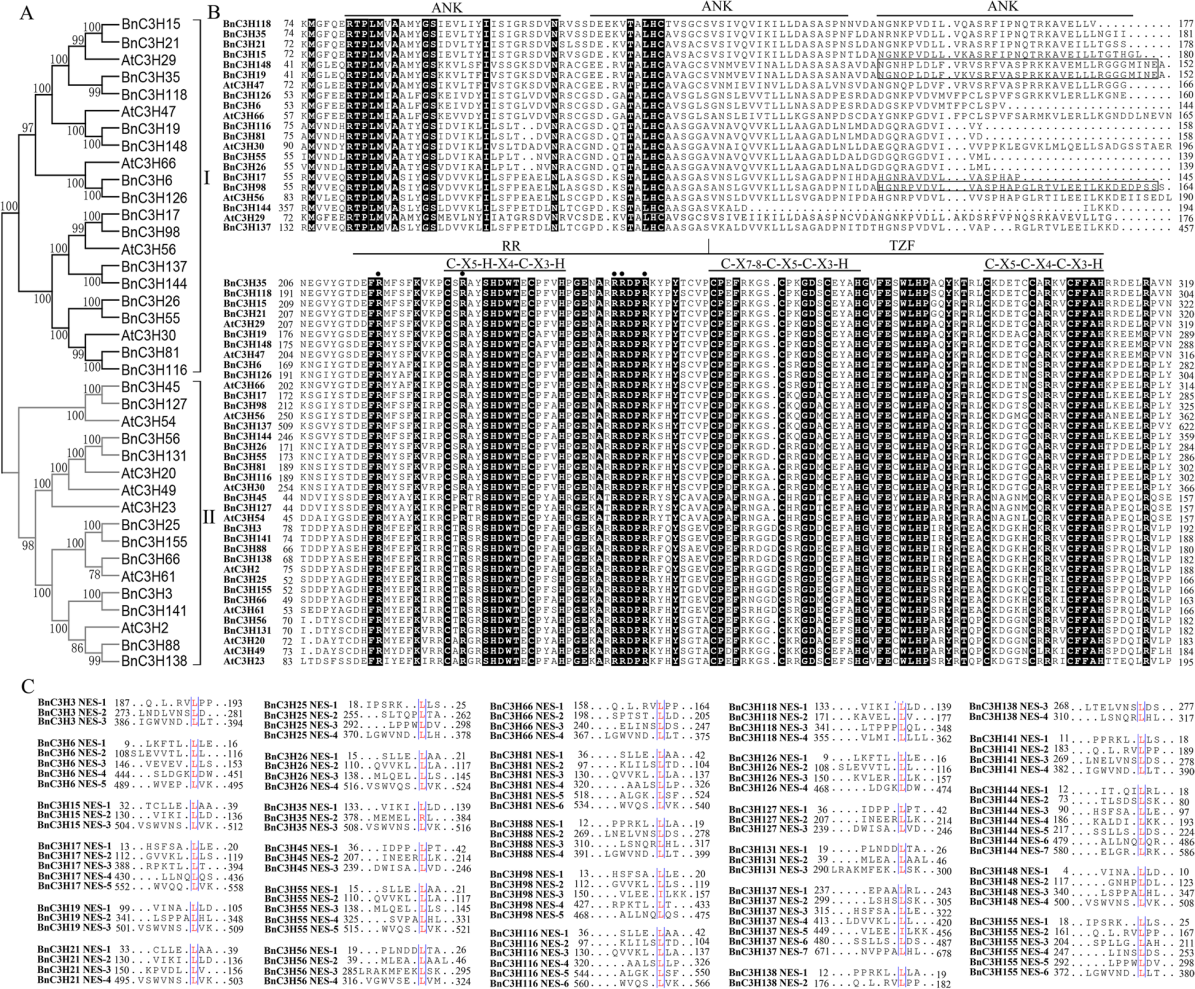
Protein sequence analysis of subfamily-IX in *B. napus*. **A**, Phylogenetic tree of subfamily-IX proteins in Arabidopsis-*B. napus* (Statistical Method: Neighbor-joining; Model/Method: p-distance; Gaps/Missing Data Treatment: Complete deletion; No. of Bootstrap Replications: 1000). **B**, Multiple sequence alignment of subfamily-IX proteins in Arabidopsis-*B. napus*. **C**, Amino acid sequence alignment of putative NES sequences of subfamily IX in *B. napus*

It is similar to Arabidopsis that RR-TZF proteins of *B. napus* can be divided into two groups (Fig. 7A), group I ANK-RR-TZF including 16 members, and group II RR-TZF including 11 members (Fig. 7B). ANK (Ankyrin) protein would be involved in responding to various biotic and abiotic stresses and regulating the growth and development of plants. *B. napus* RR-TZF proteins contain two conserved motifs, C-X_7–8_-C-X_5_-C-X_3_-H and C-X_5_-C-X_4_-C-X_3_-H spaced by 16 amino acids (TZF) and an arginine-rich motif (RR) which contains a conserved C-X_5_-H-X_4_-C-X_3_-H motif in front of the TZF domain (Fig. 7B). In animals, TTP was translocated from the nucleus mediated by a Leucine-rich Nuclear Export Signal (NES). 117 NES sequences were identified from 27 members of subfamily IX. The result suggests that all subfamily IX proteins of *B. napus* may be nucleocytoplasmic shuttle proteins involved in signal transduction (Fig. 7C).

### Stress response of subfamily IX in *B. napus*

**Fig. 8:**
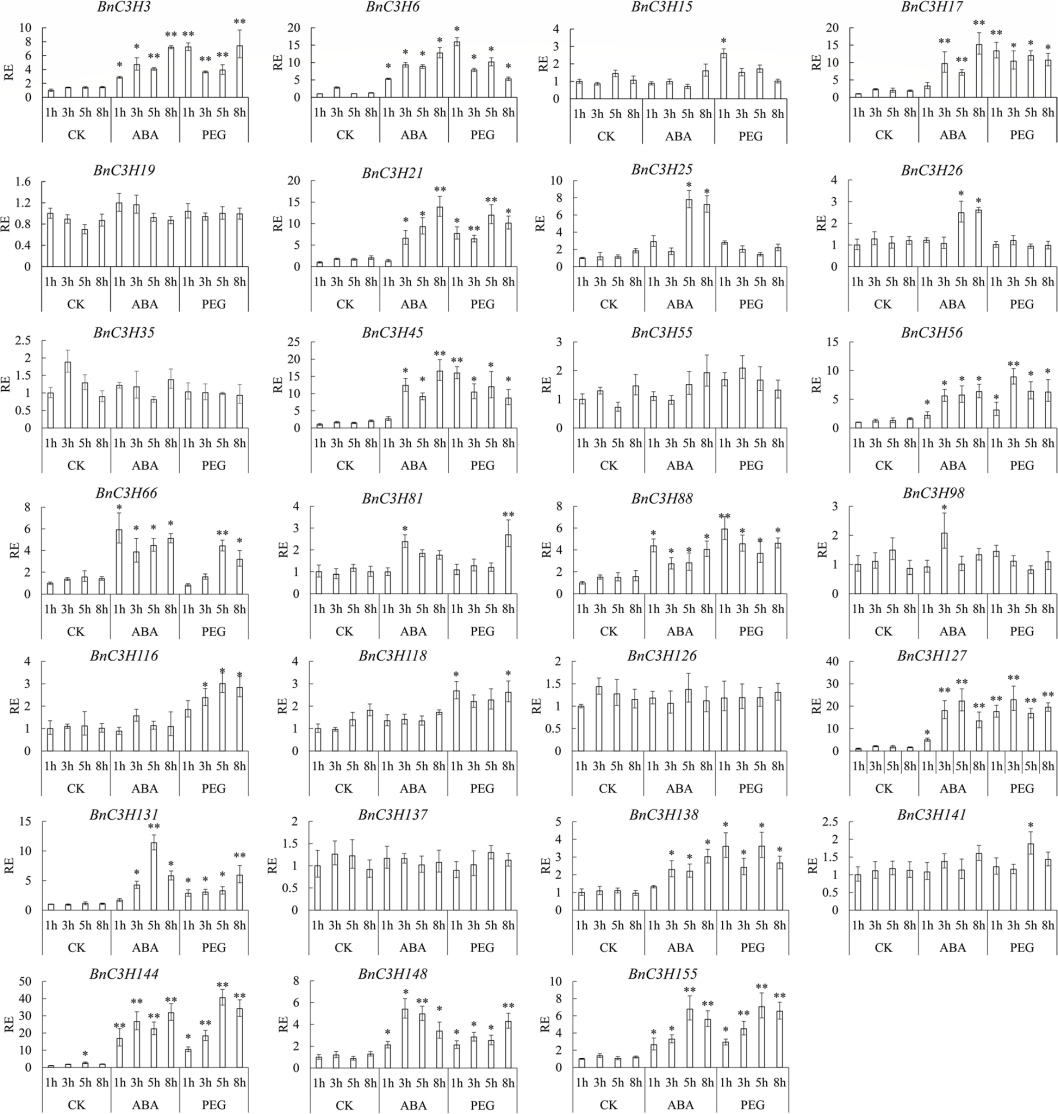
Expression profiles of *CCCH* genes of subfamily-IX in response to ABA and PEG treatment. The relative expression of *BnRR-TZF*s in *B.napus* under 100 μM ABA, 25% PEG (drought) conditions. The data are representative of three independent experiments (*n* = 3, mean ± SD, *p < 0.05, **p < 0.01, t.test)

A total of 27 *RR-TZF* genes in subfamily IX, 14 of them belong to subgenome A and 13 of them belong to subgenome C. To study the response of subfamily IX genes in abiotic stresses ABA and drought, the expression of *RR-TZF* genes under ABA or PEG conditions was verified by qRT-PCR at four different time points. The results showed that 22 of 27 *BnRR-TZF* genes were able to respond to ABA or PEG stress (Fig. 8). Among them, the expression of 9 *CCCH* genes *(BnC3H3, BnC3H6, BnC3H21, BnC3H56, BnC3H88, BnC3H127, BnC3H144, BnC3H148* and *BnC3H155)* was quickly elevated by ABA and PEG treatment and maintained higher than that of the control at all examined time points. In subfamily IX, ten diploid duplication gene pairs and two triploid duplication gene groups were found. And all gene pairs occurred segmental duplication. Among duplication gene pairs, a part of gene pairs may be functionally conserved so they have similar expression patterns. For example, *BnC3H56* and *BnC3H131*, a pair of diploid duplication gene pairs homologous to *AtTZF2* (*AtC3H20*), were notably up-regulated by ABA and PEG [37, 38] (Figs. 7 and 8). A part of *BnRR-TZF* genes only response to ABA or PEG treatment. *BnC3H25, BnC3H26* and *BnC3H98* were the only ABA-induced genes. Also, some gene pairs have different structures, which may contribute to functional diversity. A group of diploid duplication gene pairs homologous to *AtTZF8 (AtC3H56)*, *BnC3H17* and *BnC3H98*, their expression pattern was different. *BnC3H98* possesses one more ANK motif than *BnC3H17*, but it just response to ABA at 3 h whereas *BnC3H17* responds both to ABA and PEG treatment (Figs. 5 and 8). Additionally, 4 genes (*BnC3H15, BnC3H116, BnC3H118* and *BnC3H141)* was only response to PEG treatment. *AtTZF10* (*AtC3H29*) was induced by salt to enhance mRNA accumulation and had nothing to ABA [39]. *BnC3H15*, *BnC3H35* and *BnC3H118,* a group of triploid duplication gene pairs, were homologous to *AtC3H29*, they have similar protein structures, but functions diversity. Our results showed that *BnC3H15* and *BnC3H118* responded fast to PEG, but not to ABA whereas *BnC3H35* had no response to ABA and PEG (Fig. 8).

In *B. napus*, most of the RR-TZF genes that responded to ABA and PEG showed extreme differences around treatment 3-5 h. Some genes showed significant changes in expression around 1 h under ABA and PEG treatment. While over time, transcripts of most genes were gradually stabilized and some even showed a downward trend, with only the highest expression at a certain point in time. There is another part of genes that are not induced by ABA and PEG, which may not be involved in the stress response to ABA and PEG induction (Fig. 8).

## Discussion

### CCCH is a large transcription factor family in *B. napus*

CCCH families have been identified in several plant species. *B. napus* is an allotetraploid species that experienced extensive genome duplication and merging events [30]. A total of 155 putative CCCH protein-encoding genes in *B. napus* were identified (Additional file 1). The CCCH proteins in *B. napus* are much more than some species based on other reports (Additional file 4), 68 in Arabidopsis (2n = 10), 67 in rice (2n = 24) [19], 68 in maize (2n = 20) [40], 91 in poplar (2n = 38) [12], 103 in switchgrass (2n = 18) [22], 89 in banana (3n = 33) [23], but less than the sum of its parents *B. rapa* (103) and *B. oleracea* (75) [21]. The genome size of *B. napus* (AACC, 2n = 38) is 1132 Mb [41], which is less than the sum of its diploid ancestors *B. rapa* (AA, 2n = 20, 485 Mb) [42] and *B. oleracea* (CC, 2n = 18, 648 Mb) [43]. Previous study shows that most orthologous gene pairs in *B. rapa* and *B. oleracea* remain as homeologous pairs in *B. napus*, whereas the occurrence of gene deletion in its parents probably predated the allopolyploidization of *B. napus* [44]. That’s probably why the number of BnC3H proteins was less than the sum of its parents.

*B. rapa* provides A-subgenome for *B. napus.* Transcription factors of *B. rapa*, respond to important environmental factors (salt, cold, osmotic stress, light, wounding, pathogen defense, cadmium and zinc ions) and plant hormones (jasmonic acid, auxin, salicylic acid, ethylene, brassinosteroid, cytokinin, and abscisic acid) are over-retained [42]. Genome polyploidization may have extended to gene families and serve as a basis to cope with extreme environments [36]. Whole genome duplication (WGD) and polyploidy events might have contributed to the CCCH number increased in the *Brassica* species [30, 45]. Whole-genome sequences showed that *B. rapa* transcription factors underwent diploidization and triploidization [42]. *B. napus* CCCH transcription factors might be over-retained as well as deletion (Fig. 2; Additional file 1).

### The evolution and conservation of CCCH proteins in *B. napus*

Gene structure, domain organization and phylogenetic tree showed that CCCH is relatively conserved in plants. Similar to the model plants Arabidopsis and rice [19] and its parent *B. rapa* [21], introns/exons of *BnC3H* genes change in a wide range, from1–18, but much conservation in the same subfamily (Fig. 4). Among duplicated gene pairs, paralogues also showed many similarities in gene structures and domain organization (Figs. 4 and 5, Additional file 3). The similarities indicate similar functions [23].

CCCH motifs were normal in plant species. The C-X_8_-C-X_5_-C-X_3_-H and C-X_7_-C-X_5_-C-X_3_-H types of motifs are predominant motifs in the CCCH protein family of *B. napus*, and the ratio is 64% and 24%, respectively (Fig. 5, Additional file 4). As Zhuang [46] indicated that overexpression *PdC3H17* can enhance the ability to remove reactive oxygen species (ROS), thereby enhancing salt tolerance depends on its CCCH domains. Thus, these CCCH motifs existed both in monocots and dicots and might play vital functions as a transcriptional binding site in abiotic stress. Compared with the dicotyledon model plant Arabidopsis, the C-X_17_-C-X_5_-C-X_3_-H motif was found in *B. napus*, but C-X_7/8_-C-X_6_-C-X_3_-H and C-X_9_-C-X_5_-C-X_3_-H motifs were disappeared. And compared with monocotyledon model plant rice, the C-X_17_-C-X_5_-C-X_3_-H motif was also found, but C-X_15_-C-X_5_-C-X_3_-H and C-X_8_-C-X_5_-C-X_4_-H were disappeared [19]. Compared with *B. rapa*, one parent of *B. napus*, there are six CCCH motifs (C-X_7/8_-C-X_6_-C-X_3_-H, C-X_12/14_-C-X_5_-C-X_3_-H, C-X_8_-C-X_5_-C-X_2_-H and C-X_9_-C-X_5_-C-X_3_-H) were not found, but the C-X_6_-C-X_6_-C-X_3_-H motif was discovered [21]. Except CCCH motifs, the TIR domain, a toll/interleukin receptor involved in relative processes of innate immunity pathways [47] and signal transduction [48], was found in subfamily XIV in *B. napus* but not in Arabidopsis and *B. rapa*. It suggests that subfamily XIV *BnC3H* might be neofunctionalization during the evolution process and play roles in innate immunity and signal transduction. Besides, RING [49], RRM [50], ANK [51], WD40 [52] and KH domain [53] are detected (Fig. 5). These motifs are related to protein-protein or protein-DNA or RNA binding in plants [54].

### Putative cis-elements and motif indicating stress response of *RR-TZF* in *B. napus*

Transcription factors activated by biotic and abiotic stresses initiated the expression of corresponding genes by binding to related elements. Abundant hormone-responsive, stress-responsive and light-responsive elements exist in all promoters of *RR-TZF* homologous genes of *B. napus* (Additional file 5, Fig. 6). Overexpression of *OsC3H10* that carries three DREs and two ABREs (ABA response element) in its promoter improved drought tolerance in rice by regulating drought-induced *OsDREB2* transcription factors through ABA-independent pathway [17]. Arabidopsis *AtTZF1*, *AtTZF2*, and *AtTZF3* equipped with ABRE (ABA response element), SARE (SA response element), TCA-element (MeJA response element) in their promoters respond to ABA, drought, oxygen, and salt stress [37, 55]. Homologous to *AtTZF2, BnC3H56* and *BnC3H131* have ABRE, ARE, MYB, CGTCA and TGACG motifs in their promoter (Additional file 5). The expression of ANK-RR-TZF subfamily genes in Arabidopsis (*AtTZF1–6*) might have evolved from the pre-existing pathways that regulate ABA-mediated responses to salt stress during the germination process [56]. The MYB binding sites function as cis-acting elements in the dehydration-induced expression of *RD22* in Arabidopsis [57]. Thus, *BnC3H88* and *BnC3H138* might enhance drought stress through MYB binding site located in its promoter by an ABA-dependent pathway. Comparing with Arabidopsis, a variety of cis-elements were detected in subfamily IX of *B. napus* CCCH family, and it showed that differentiation event might have occurred in BnRR-TZF to a certain extent during the *CCCH* gene family evolution process. It suggests that *RR-TZF* genes may play a crucial role in response to hormone-induced and abiotic stresses.

Tandem CCCH Zinc Finger proteins (TZFs) are conserved from yeast to metazoans [16]. In animals, the structure of CCCH type TZF domain has been determined from the TIS11D [58] and the AU-rich element from the 3′-UTR of TNF-α transcript as a binding partner of the TZF domain [59]. Different from yeast and metazoans, plant TZF motif was conserved preceded by arginine-rich (RR) domains. Similar to Arabidopsis, subfamily IX of *B. napus CCCH* gene family were divided into two groups, group I charactered with RR-TZF domain and extra two or three ANK domains, group II charactered with RR-TZF domain (Fig. 7). The Nuclear Export Signal (NES) of subfamily IX protein infers that the *BnRR-TZF* might be involved in signal transduction [19] (Fig. 7). The function of all members of *RR-TZFs* related to biotic and abiotic stresses in Arabidopsis was summed up. Because of the conservation of TZF domain in evolution, plant RR-TZF domain might have a similar mechanism to animals TZF on RNA targeting and transcriptional regulation in stress response.

### *RR-TZF* genes involving stress response in *B. napus*

Arabidopsis *RR-TZF* genes are involved in ABA, salt, cold, H_2_O_2_, osmotic, and sugar depletion stresses [16]. The expression patterns of abiotic stress responsive show that more than half of *B. napus RR-TZF* can be induced by ABA and PEG (Fig. 8). *AtC3H23(AtTZF1), AtC3H20 (AtTZF2) and AtC3H49 (AtTZF3)* located in cytoplasm and expressed in vegetative tissues and flowers, and overexpression *AtTZF1*, *AtTZF2,* or *AtTZF3* caused ABA hypersensitivity, enhanced drought tolerance and reduced transpiration [37, 38]. *BnC3H45, BnC3H56, BnC3H127* and *BnC3H131*, homologous genes of *AtTZF1*, *AtTZF2* or *AtTZF3*, responded to the induction of ABA and PEG stress. In particular *BnC3H127*, always kept a high expression level under ABA and PEG stress. *AtTZF4*, *AtTZF5*, *AtTZF6* were much important to regulate seed germination in Arabidopsis [25, 60]. And part of their homologous genes in *B. napus BnC3H3, BnC3H66, BnC3H88, BnC3H138* and *BnC3H155* were also responded ABA and PEG (Fig. 8). Double mutant *tzf10 tzf11* is more sensitive to salt stress and drought stress in Arabidopsis [61]. Homologous genes of *AtC3H29 (AtTZF10)* and *AtC3H47 (AtTZF11)* in *B. napus BnC3H15, BnC3H21, BnC3H118, BnC3H35, BnC3H19* and *BnC3H148* might undergo function diversification. *BnC3H15/BnC3H21/BnC3H118*/*BnC3H148* responded to PEG, while *BnC3H21* and *BnC3H148* responded to ABA. These four *RR-TZF* genes belong to the ANK-RR-TZF subfamily, and they have similar gene and protein structures. Furthermore, *BnC3H118* lacked ABA-related elements (Fig. 6). *BnC3H15,* a homologous gene to *AtTZF10,* has an ABRE like *BnC3H21* and *BnC3H148*, but only responds to PEG but not ABA. The results of gene expression (Fig. 8) and the stress-responsive cis-elements in *BnRR-TZFs* promoters (Fig. 6) suggest that some *BnRR-TZFs* respond to hormones (like ABA, JA, etc.), drought and salty treatments.

Most of the time, duplication genes have a similar expression pattern which one is from subgenome A and another is from subgenome C [62]. This kind of duplicated genes may be functionally conserved. But some duplicated genes diversify in response to ABA and PEG in *BnRR-TZFs*. During the evolution of *CCCH* gene family in *B. napus*, new neofunctionalization appeared [20]. This phenomenon may have occurred in CCCH family in *B. napus*. Different structures may lead to different duplication types and functional differences [63]. In BnRR-TZF subfamily, a diploid duplication gene pairs *BnC3H17* and *BnC3H98*, their protein structures were different, they responded differently to ABA and PEG. It may indicate that some functional divergence has occurred to the duplication genes in BnRR-TZF family.

RR-TZF proteins trigger mRNA degradation by binding to 3′-UTR of target mRNAs in a sequence-specific manner [16, 64]. But stress-responsive target genes activated by RR-TZF proteins have not been confirmed in plants. It is inferred that *BnRR-TZF* genes might respond to ABA and drought stress in a similar way to Arabidopsis because of their close relationship, similar cis-acting elements in the promoter region and conservative domain organization (Figs. 3, 5 and 6). Identification of the target genes or mRNAs of CCCH proteins, understanding the mechanism of binding and activation between CCCH protein and target gene or mRNA are worth further analyzing.

## Materials and methods

### Characterization and identification of CCCH proteins in *Brassica napus*

To identify CCCH proteins of *B. napus*, the genome sequence of Arabidopsis, rice, *B. rapa* and *B. oleracea* are cited from references [19, 21], and the *CCCH* genes and proteins sequence of *B. napus* were obtained from the Genome Resources database (http://www.genoscope.cns.fr/brassicanapus/) by using the Basic Local Alignment Search Tool algorithms program (BLASTP) with Arabidopsis CCCH protein sequences as queries. Further, the candidate sequences were confirmed by SMART website (http://smart.embl-heidelberg.de/) and NCBI conserved domain search tools (https://www.ncbi.nlm.nih.gov/Structure/cdd/wrpsb.cgi).

The chromosome location information of *BnC3H* genes was subjected to MapChart 2.2 software to draw the draft [65]. The physicochemical parameters of BnC3H proteins were generated by the program ExPASy (https://web.expasy.org/protparam/).

### Genomic organization and syntenic analysis of *CCCH* in *B. napus*

To visualize the location and syntenic gene pairs of *BnC3H* in genome, the gene position, gene length, chromosome size, and centromere position were extracted from the gff files of *B. napus* genome (https://www.genoscope.cns.fr/brassicanapus/data/). All protein sequences of *B. napus* were compared against themselves, and the distribution map was drawn by the MCScanX tool on TBtools software (E-value < 1e^− 5^, number of hit≤5) [66].

### Gene duplication, Ka/Ks calculation and selection pressure analysis

The duplicated gene groups were defined as the methods of Yang [33] and tandem duplicated groups were defined as the methods of Sun [67]. The full-length-CDS sequence covering and identify of amino acid were detected by Blastn/Blastp in NCBI [68].

The non-synonymous substitution rate (Ka), synonymous substitution rate (Ks), and the duplication time (T, million years ago, MYA) were calculated by a Simple Ka/Ks Calculator tool on TBtools software [66]. The selection pressure on *BnC3H* duplicated gene groups were detected through Ka/Ks ratio and considered positive, negative or neutral selection when Ka/Ks ratio was> 1, < 1, or = 1, respectively [32].

### Analysis of gene structure, domain organization, and phylogenetic relationship

To further understand the structural features of *BnC3H* genes, we deduced the exon-intron organization map by comparing cDNA with their corresponding genomic sequences of *BnC3H*. After genomic and cDNA sequences were downloaded from the *B. napus* database (http://www.genoscope.cns.fr/brassicanapus/), the gene structure was constructed by the Gene Structure Display Server (http://gsds.gao-lab.org/index.php) [69]. The information of domain organization was identified by SMART and Conserved Domain Search tool on NCBI (https://www.ncbi.nlm.nih.gov/Structure/cdd/wrpsb.cgi), then the sites of domain organization were constructed by IBS 1.0.3 software [70].

To explore the phylogenetic relationship of BnC3H, we have constructed a phylogenetic tree including 68 AtC3H, 67 OsC3H, 103 BraC3H, 75 BolC3H and 155 BnC3H proteins. Multiple sequence alignment of BnC3H proteins was carried out using the MUSCLE (Multiple Sequence Comparison by Log- Expectation) programs [71] and the resulting file was subjected to phylogenic analysis using the MEGA 7.0 program [72]. A tree was constructed based on the full-length protein sequences using the Maximum Likelihood (ML) method with Poisson model, and a Bootstrap test of 1000 replicates for internal branch reliability.

The conserved domain of ANK and RR-TZF in subfamily IX were isolated from CCCH zinc finger proteins by ESPript3.0 website (http://espript.ibcp.fr/ESPript/ESPript/index.php), the Nuclear Export Signal (NES) sequences were detected with a program as Wang [19], and the draft files were also created by ESPript3.0 wedsite.

### Prediction of BnRR-TZF promoter cis-acting element

To identify the cis-acting element of subfamily IX in *B. napus*, an upstream 1500 bp promoter sequence of the *CCCH* gene start codon was extracted to predict their putative cis-element by PlantCARE (http://bioinformatics.psb.ugent.be/webtools/ plantcare/html/) [73].

### Plant materials and stress treatment

*B. napus* Xiang You 15 (XY15) was used as plant material. It was bred from Hunan Agricultural University (Changsha, China) and stored in the Key Laboratory of Crop Epigenetic Regulation and Development in Hunan Province. XY15 seeds grew in roseite in the green room at 24 °C and 70% humidity with 16 h light/8 h dark photoperiod. Three-week-old seedlings with 2–3 true leaves were cleaned up and cultivated in 1/2 liquid MS medium in a growth chamber for 3 days to acclimatize before treatment with ABA and PEG. The whole seedlings were harvested and put into 1/2 liquid MS medium with 100 μM ABA or 25% PEG. The seedlings were sampled to detect *CCCH* gene expression at 1, 3, 5, and 8 h at the process. Seedlings in 1/2 liquid MS medium were used as control at the same time points. Triplicate seedling samples were collected and quickly frozen in liquid nitrogen and then stored at − 80 °C [74, 75]. Triplicate was confirmed.

### Quantitative real-time PCR (qRT-PCR) validation

RNA isolation of *B. napus* were carried out by TRIzol reagent kit (Invitrogen, Carlsbad, CA, US) according to the instructions. The quality of RNA was determined using a NanoDrop 2000 spectrophotometer (ThermoFisher Scientific, USA), and the integrity was evaluated using agarose gel electrophoresis stained with ethidium bromide. Approximately 1.0 μg total RNA was reverse-transcribed into cDNA using an RT reagent kit (RevertAid Fitst Strand cDNA Synthesis, ThermoFisher Scientific, USA) [68].

The quantitative real-time PCR was carried out with SYBR-green fluorescence using a CFX 96 Real-Time System (BIO-RAD) with a 20 μl PCR reaction mixture that included 8.8 μl of 10 decuple diluted cDNA, 10 μl of 2 × FastStart Universal SYBR Green Master (ROX) (Roche, Switzerland), and 10 mM 0.6 μl of forward and reverse primer as previously. The *BnaA10g22340D* gene was used as a reference gene [76]. All primer was designed by NCBI primer blast tools (Additional file 6). Each sample was run in triplicate for analysis. At the end of the PCR cycles, the melting curve analysis was performed to validate the specific generation of the expected PCR product. The expression levels of *BnRR-TZF* genes were calculated with 2^−ΔΔCT^ method as a previous report [77].

## Conclusion

Allotetraploid *B. napus* inherited *CCCH* genes from its diploid parents *B. rapa* and *B. oleracea*, and its genome has undergone multiple duplications and deletions. 155 *CCCH* genes, 81 from subgenome A and 74 from subgenome C were identified. Evolutionary relationship, gene and protein structure analysis in CCCH family in *B. napus* showed diversity among subfamilies, but highly conservation within the same subfamily. Subfamily-IX *RR-TZF* genes are involved in ABA or drought stress. The results presented basic information of *BnC3H* genes and provided a useful resource for gene function and breeding of Brassicase crops.

## Supplementary Information

**Additional file 1.** (XLS 272 kb)

**Additional file 2.** (XLS 4288 kb)

**Additional file 3.** (XLS 128 kb)

**Additional file 4.** (XLS 32 kb)

**Additional file 5.** (XLS 53 kb)

**Additional file 6.** (XLS 31 kb)

## Data Availability

All data generated or analyzed during this study are obtained from published articles and their Additional files. The protein sequences of CCCH family genes in *B. napus* were downloaded from https://www.genoscope.cns.fr/brassicanapus/. All sequences were identified by using the online protein structure prediction tool SMART (http://smart.embl-heidelberg.de/). The supporting data of this article are included in the article and its Supplementary files. In this article, the collection and cultivation of plant material comply with relevant institutional, national, and international guidelines and legislation.

## Abbreviations

ABRE: ABA response element
ABA: Abscisic acid
ANK: Ankyrin
AP2/ERF: APETALA2/Ethylene-Responsive Factor
RR-TZF: Arginine rich-tandem CCCH-type zinc finger
bHLH: Basic Helix–Loop–Helix
bZIP: Basic Leucine Zipper
BLASTP: Basic Local Alignment Search Tool algorithms Program
C3H: CCCH
*DGAT1*: Diacylglycerol O-acyltransferase 1
HD-ZIP: Homeoomain-leucine Zipper
JA: Jasmonic acid
KH: K-Homology
ML: Maximum Likelihood
MYA: Million years ago
MS: Murashige and Skoog
MUSCLE: Multiple Sequence Comparison by Log- Expectation
Ka: Non-synonymous substitution rate
non-TZF: Non-tandem CCCH-type zinc finger
NES: Nuclear Export Signal
PEG: Polyethylene glycol
ROS: Reactive oxygen species
RRM: RNA recognition motif
SARE: SA response element
SMART: Simple Modular Architecture Research Tool
Ks: Synonymous substitution rate
TZF: Tandem CCCH-type zinc finger
MeJA: Methyl Jasmonate
TIR: Toll/interleukin receptor
TFs: Transcription factors
TTP: Tristetraprolin
UTR: Untranslated Region
WD40: WD or beta-transducin repeats
WGD: Whole genome duplication
ZFP: Zinc Finger Protein

## References

[1] Banerjee A, Roychoudhury A (2017). Abscisic-acid-dependent basic leucine zipper (bZIP) transcription factors in plant abiotic stress. Protoplasma..

[2] Agarwal P, Baranwal VK, Khurana P (2019). Genome-wide analysis of bZIP transcription factors in wheat and functional characterization of a *TabZIP* under abiotic stress. Sci Rep-Uk.

[3] Phukan UJ, Jeena GS, Shukla RK (2016). WRKY transcription factors: molecular regulation and stress responses in plants. Front Plant Sci.

[4] Guo B, Wei Y, Xu R, Lin S, Luan H, Lv C, Zhang X, Song X, Xu R (2016). Genome-wide analysis of APETALA2/ethylene-responsive factor (AP2/ERF) gene family in barley *(Hordeum vulgare* L.). PLoS One.

[5] Cui MH, Yoo KS, Hyoung S, Nguyen HTK, Kim YY, Kim HJ, Ok SH, Yoo SD, Shin JS (2013). An Arabidopsis R2R3-MYB transcription factor, *AtMYB20*, negatively regulates type 2C serine/threonine protein phosphatases to enhance salt tolerance. FEBS Lett.

[6] Hoang XLT, Nhi DNH, Thu NBA, Thao NP, Tran LSP (2017). Transcription factors and their roles in signal transduction in plants under abiotic stresses. Curr Genomics.

[7] Wang YX, Liu ZW, Wu ZJ, Li H, Zhuang J (2016). Transcriptome-wide identification and expression analysis of the NAC gene family in Tea plant [*Camellia sinensis* (L.) O. Kuntze]. PLoS One.

[8] Li P, Zhang B, Su TB, Li PR, Xin XY, Wang WH, Zhao XY, Yu YJ, Zhang DH, Yu SC (2018). BrLAS, a GRAS transcription factor from Brassica rapa, is involved in drought stress tolerance in transgenic Arabidopsis. Front Plant Sci.

[9] He X, Wang TY, Zhu W, Wang YJ, Zhu LF (2018). GhHB12, a HD-ZIP I transcription factor, negatively regulates the cotton resistance to *Verticillium dahliae*. Int J Mol Sci.

[10] Kielbowicz-Matuk A (2012). Involvement of plant C2H2-type zinc finger transcription factors in stress responses. Plant Sci.

[11] Han GL, Lu CX, Guo JR, Qiao ZQ, Sui N, Qiu NW, Wang BS (2020). C2H2 zinc finger proteins: master regulators of abioticstress responses in plants. Front Plant Sci.

[12] Chai GH, Hu RB, Zhang DY, Qi G, Zuo R, Cao YP, Chen P, Kong YZ, Zhou GK (2012). Comprehensive analysis of CCCH zinc finger family in poplar (*Populus trichocarpa*). BMC Genomics.

[13] Seok HY, Nguyen LV, Park HY, Tarte VN, Ha J, Lee SY, Moon YH (2018). Arabidopsis non-TZF gene *AtC3H17* functions as a positive regulator in salt stress response. Biochem Biophys Res Commun.

[14] Seok HY, Woo DH, Park HY, Lee SY, Tran HT, Lee EH, Vu Nguyen L, Moon YH (2016). *AtC3H17*, a non-tandem CCCH zinc finger protein, functions as a nuclear transcriptional activator and has pleiotropic effects on vegetative development, flowering and seed development in Arabidopsis. Plant Cell Physiol.

[15] Zhang H, Zhang Z, Xiong T, Xiong X, Wu X, Guan C, Xiao G (2018). The CCCH-type transcription factor *BnZFP1* is a positive regulator to control oleic acid levels through the expression of diacylglycerol O-acyltransferase 1 gene in Brassica napus. Plant Physiol Biochem.

[16] Jang JC (2016). Arginine-rich motif-tandem CCCH zinc finger proteins in plant stress responses and post-transcriptional regulation of gene expression. Plant Sci.

[17] Seong SY, Shim JS, Bang SW, Kim JK (2020). Overexpression of *OsC3H10*, a CCCH-zinc finger, improves drought tolerance in Rice by regulating stress-related genes. Plants (Basel).

[18] Qu J, Kang SG, Wang W, Musier-Forsyth K, Jang JC (2014). The Arabidopsis thaliana tandem zinc finger 1 (AtTZF1) protein in RNA binding and decay. Plant J.

[19] Wang D, Guo Y, Wu C, Yang G, Li Y, Zheng C (2008). Genome-wide analysis of CCCH zinc finger family in Arabidopsis and rice. BMC Genomics.

[20] Cannon SB, Mitra A, Baumgarten A, Young ND, May G (2004). The roles of segmental and tandem gene duplication in the evolution of large gene families in *Arabidopsis thaliana*. BMC Plant Biol.

[21] Pi B, He X, Ruan Y, Jang JC, Huang Y (2018). Genome-wide analysis and stress-responsive expression of CCCH zinc finger family genes in *Brassica rapa*. BMC Plant Biol.

[22] Yuan SX, Xu B, Zhang J, Xie ZN, Cheng Q, Yang ZM, Cai QS, Huang BR (2015). Comprehensive analysis of CCCH-type zinc finger family genes facilitates functional gene discovery and reflects recent allopolyploidization event in tetraploid switchgrass. BMC Genomics.

[23] Mazumdar P, Lau S-E, Wee WY, Singh P, Harikrishna JA (2017). Genome-wide analysis of the CCCH zinc-finger gene family in Banana (*Musa acuminata*): an insight into motif and gene structure arrangement, evolution and salt stress responses. Trop Plant Biol.

[24] Chen F, Liu HL, Wang K, Gao YM, Wu M, Xiang Y (2020). Identification of CCCH zinc finger proteins family in *Moso bamboo* (*Phyllostachys edulis*), and *PeC3H74* confers drought tolerance to transgenic plants. Front Plant Sci.

[25] Bogamuwa S, Jang JC (2013). The Arabidopsis tandem CCCH zinc finger proteins *AtTZF4, 5* and *6* are involved in light-, abscisic acid- and gibberellic acid- mediated regulation of seed germination. Plant Cell Environ.

[26] Yuan X, Zhang S, Qing X, Sun M, Liu S, Su H, Shu H, Li X (2013). Superfamily of ankyrin repeat proteins in tomato. Gene..

[27] Wells ML, Perera L, Blackshear PJ (2017). An ancient family of RNA-binding proteins: still important!. Trends Biochem Sci.

[28] Ramos SB, Stumpo DJ, Kennington EA, Phillips RS, Bock CB, Ribeiro-Neto F, Blackshear PJ (2004). The CCCH tandem zinc-finger protein *Zfp36l2* is crucial for female fertility and early embryonic development. Development..

[29] Maeda K, Akira S (2017). Regulation of mRNA stability by CCCH-type zinc-finger proteins in immune cells. Int Immunol.

[30] Di F, Jian H, Wang T, Chen X, Ding Y, Du H, Lu K, Li J, Liu L (2018). Genome-wide analysis of the *PYL* gene family and identification of *PYL* genes that respond to abiotic stress in *Brassica napus*. Genes (Basel).

[31] Arda Soylev TML, Amini H, Alkan C (2019). Fereydoun Hormozdiari. Discovery of tandem and interspersed segmental duplications using high-throughput sequencing. Bioinformatics..

[32] Ahmad MZ, Sana A, Jamil A, Nasir JA, Ahmed S, Hameed MU, Abdullah. (2019). A genome-wide approach to the comprehensive analysis of *GASA* gene family in *Glycine max*. Plant Mol Biol.

[33] Yang SH, Zhang XH, Yue JX, Tian DC, Chen JQ (2008). Recent duplications dominate NBS-encoding gene expansion in two woody species. Mol Gen Genomics.

[34] Kummerfeld SK, Teichmann SA (2009). Protein domain organisation: adding order. BMC Bioinformatics.

[35] Nardi CF, Villarreal NM, Opazo MC, Martinez GA, Moya-Leon MA, Civello PM (2014). Expression of *FaXTH1* and *FaXTH2* genes in strawberry fruit. Cloning of promoter regions and effect of plant growth regulators. Sci Hortic-Amsterdam.

[36] Peer VY, Ashman TL, Soltis PS, Soltis DE (2020). Polyploidy: an evolutionary and ecological force in stressful times. Plant Cell.

[37] Lee SJ, Jung HJ, Kang H, Kim SY (2012). Arabidopsis zinc finger proteins *AtC3H49/AtTZF3* and *AtC3H20/AtTZF2* are involved in ABA and JA responses. Plant Cell Physiol.

[38] Huang P, Ju HW, Min JH, Zhang X, Chung JS, Cheong HS, Kim CS (2012). Molecular and physiological characterization of the Arabidopsis thaliana oxidation-related zinc finger 2, a plasma membrane protein involved in ABA and salt stress response through the ABI2-mediated signaling pathway. Plant Cell Physiol.

[39] Blanvillain R, Wei S, Wei PC, Kim JH, Ow DW (2011). Stress tolerance to stress escape in plants: role of the *OXS2* zinc-finger transcription factor family. EMBO J.

[40] Peng XJ, Zhao Y, Cao JG, Zhang W, Jiang HY, Li XY, Ma Q, Zhu SW, Cheng BJ (2012). CCCH-type zinc finger family in maize: genome-wide identification, classification and expression profiling under abscisic acid and drought treatments. PLoS One.

[41] Johnston JS, Pepper AE, Hall AE, Chen ZJ, Hodnett G, Drabek J, Lopez R, Price HJ (2005). Evolution of genome size in Brassicaceae. Ann Bot.

[42] Wang XWH, Wang J, Sun R, Wu J, Liu S, Bai Y, Mun JH, Bancroft I, Cheng F, Huang S, Li X, Hua W, Wang J, Wang X, Freeling M, Pires JC, Paterson AH, Chalhoub B, Wang B, Hayward A, Sharpe AG, Park BS, Weisshaar B, Liu B, Li B, Liu B, Tong C, Song C, Duran C, Peng C, Geng C, Koh C, Lin C, Edwards D, Mu D, Shen D, Soumpourou E, Li F, Fraser F, Conant G, Lassalle G, King GJ, Bonnema G, Tang H, Wang H, Belcram H, Zhou H, Hirakawa H, Abe H, Guo H, Wang H, Jin H, Parkin IA, Batley J, Kim JS, Just J, Li J, Xu J, Deng J, Kim JA, Li J, Yu J, Meng J, Wang J, Min J, Poulain J, Wang J, Hatakeyama K, Wu K, Wang L, Fang L, Trick M, Links MG, Zhao M, Jin M, Ramchiary N, Drou N, Berkman PJ, Cai Q, Huang Q, Li R, Tabata S, Cheng S, Zhang S, Zhang S, Huang S, Sato S, Sun S, Kwon SJ, Choi SR, Lee TH, Fan W, Zhao X, Tan X, Xu X, Wang Y, Qiu Y, Yin Y, Li Y, Du Y, Liao Y, Lim Y, Narusaka Y, Wang Y, Wang Z, Li Z, Wang Z, Xiong Z, Zhang Z (2011). The genome of the mesopolyploid crop species *Brassica rapa*. Nat Genet.

[43] Parkin IA, Koh C, Tang H, Robinson SJ, Kagale S, Clarke WE, Town CD, Nixon J, Krishnakumar V, Bidwell SL, Denoeud F, Belcram H, Links MG, Just J, Clarke C, Bender T, Huebert T, Mason AS, Pires JC, Barker G, Moore J, Walley PG, Manoli S, Batley J, Edwards D, Nelson MN, Wang X, Paterson AH, King G, Bancroft I, Chalhoub B, Sharpe AG (2014). Transcriptome and methylome profiling reveals relics of genome dominance in the mesopolyploid *Brassica oleracea*. Genome Biol.

[44] Chalhoub B, Denoeud F, Liu S, Parkin IA, Tang H, Wang X, Chiquet J, Belcram H, Tong C, Samans B, Corréa M, Da Silva C, Just J, Falentin C, Koh CS, Le Clainche I, Bernard M, Bento P, Noel B, Labadie K, Alberti A, Charles M, Arnaud D, Guo H, Daviaud C, Alamery S, Jabbari K, Zhao M, Edger PP, Chelaifa H, Tack D, Lassalle G, Mestiri I, Schnel N, Le Paslier MC, Fan G, Renault V, Bayer PE, Golicz AA, Manoli S, Lee TH, Thi VH, Chalabi S, Hu Q, Fan C, Tollenaere R, Lu Y, Battail C, Shen J, Sidebottom CH, Wang X, Canaguier A, Chauveau A, Bérard A, Deniot G, Guan M, Liu Z, Sun F, Lim YP, Lyons E, Town CD, Bancroft I, Wang X, Meng J, Ma J, Pires JC, King GJ, Brunel D, Delourme R, Renard M, Aury JM, Adams KL, Batley J, Snowdon RJ, Tost J, Edwards D, Zhou Y, Hua W, Sharpe AG, Paterson AH, Guan C, Wincker P (2014). Early allopolyploid evolution in the post-Neolithic *Brassica napus* oilseed genome. Science..

[45] Cheng F, Wu J, Wang X (2014). Genome triplication drove the diversification of Brassica plants. Hortic Res.

[46] Zhuang Y, Wang C, Zhang Y, Chen S, Wang D, Liu Q, Zhou G, Chai G (2020). Overexpression of PdC3H17 confers tolerance to drought stress depending on its CCCH domain in *Populus*. Front Plant Sci.

[47] Nimma S, Ve T, Williams SJ, Kobe B (2017). Towards the structure of the TIR-domain signalosome. Curr Opin Struct Biol.

[48] Grech-Baran M, Witek K, Szajko K, Witek AI, Morgiewicz K, Wasilewicz-Flis I, Jakuczun H, Marczewski W, Jones JDG, Hennig J (2020). Extreme resistance to potato virus Y in potato carrying the Ry_sto_ gene is mediated by a TIR-NLR immune receptor. Plant Biotechnol J.

[49] Cano F, Rapiteanu R, Winkler GS, Lehner PJ (2015). A non-proteolytic role for ubiquitin in deadenylation of MHC-I mRNA by the RNA-binding E3-ligase MEX-3C. Nat Commun.

[50] Lee KC, Jang YH, Kim SK, Park HY, Thu MP, Lee JH, Kim JK (2017). RRM domain of Arabidopsis splicing factor *SF1* is important for pre-mRNA splicing of a specific set of genes. Plant Cell Rep.

[51] Zeng CJ, Goodluck H, Qin XZ, Liu B, Mohan S, Xing WR (2016). Leucine-rich repeat kinase-1 regulates osteoclast function by modulating *RAC1/Cdc42* small GTPase phosphorylation and activation. Am J Physiol-Endoc M.

[52] Li QH, Chang LF, Aibara S, Yang J, Zhang ZG, Barford D (2016). WD40 domain of Apc1 is critical for the coactivator-induced allosteric transition that stimulates APC/C catalytic activity. P Natl Acad Sci USA.

[53] Jeong IS, Fukudome A, Aksoy E, Bang WY, Kim S, Guan QM, Bahk JD, May KA, Russell WK, Zhu JH (2015). Regulation of abiotic stress signalling by Arabidopsis C-terminal domain phosphatase-like 1 requires interaction with a K-homology domain-containing protein. PLoS One.

[54] Sharma M, Pandey GK (2016). Expansion and function of repeat domain proteins during stress and development in plants. Front Plant Sci.

[55] Huang GT, Ma SL, Bai LP, Zhang L, Ma H, Jia P, Liu J, Zhong M, Guo ZF (2012). Signal transduction during cold, salt, and drought stresses in plants. Mol Biol Rep.

[56] D'Orso F, De Leonardis AM, Salvi S, Gadaleta A, Ruberti I, Cattivelli L, Morelli G, Mastrangelo AM (2015). Conservation of *AtTZF1, AtTZF2,* and *AtTZF3* homolog gene regulation by salt stress in evolutionarily distant plant species. Front Plant Sci.

[57] Abe H, Yamaguchi-Shinozaki K, Urao T, Iwasaki T, Hosokawa D, Shinozaki K (1997). Role of Arabidopsis *MYC* and *MYB* homologs in drought- and abscisic acid-regulated gene expression. Plant Cell.

[58] Hudson BP, Martinez-Yamout MA, Dyson HJ, Wright PE (2004). Recognition of the mRNA AU-rich element by the zinc finger domain of *TIS11d*. Nat Struct Mol Biol.

[59] Tavella D, Ertekin A, Schaal H, Ryder SP, Massi F (2020). A disorder-to-order transition mediates RNA binding of the Caenorhabditis elegans protein MEX-5. Biophys J.

[60] Kim DH, Yamaguchi S, Lim S, Oh E, Park J, Hanada A, Kamiya Y, Choi G (2008). SOMNUS, a CCCH-type zinc finger protein in Arabidopsis, negatively regulates light-dependent seed germination downstream of PIL5. Plant Cell.

[61] Maldonado-Bonilla LD, Eschen-Lippold L, Gago-Zachert S, Tabassum N, Bauer N, Scheel D, Lee J (2014). The Arabidopsis tandem zinc finger 9 protein binds RNA and mediates pathogen-associated molecular pattern-triggered immune responses. Plant Cell Physiol.

[62] Wang T, Hu JJ, Ma X, Li CJ, Yang QH, Feng SY, Li MM, Li N, Song XM (2020). Identification, evolution and expression analyses of whole genome-wide TLP gene family in *Brassica napus*. BMC Genomics.

[63] Wang YP, Tan X, Paterson AH (2013). Different patterns of gene structure divergence following gene duplication in Arabidopsis. BMC Genomics.

[64] Kim WC, Kim JY, Ko JH, Kang H, Kim J, Han KH (2014). AtC3H14, a plant-specific tandem CCCH zinc-finger protein, binds to its target mRNAs in a sequence-specific manner and affects cell elongation in *Arabidopsis thaliana*. Plant J.

[65] Wang R, Zhao P, Kong N, Lu R, Pei Y, Huang C, Ma H, Chen Q (2018). Genome-wide identification and characterization of the potato bHLH transcription factor family. Genes (Basel).

[66] Chen C, Chen H, Zhang Y, Thomas HR, Frank MH, He Y, Xia R (2020). TBtools: an integrative toolkit developed for interactive analyses of big biological data. Mol Plant.

[67] Sun R, Wang K, Guo T, Jones DC, Cobb J, Zhang B, Wang Q (2015). Genome-wide identification of auxin response factor (*ARF*) genes and its tissue-specific prominent expression in *Gossypium raimondii*. Funct Integr Genomics.

[68] Huang Y, Mo Y, Chen P, Yuan X, Meng F, Zhu S, Liu Z (2016). Identification of SET domain-containing proteins in *Gossypium raimondii* and their response to high temperature stress. Sci Rep.

[69] Hu B, Jin J, Guo AY, Zhang H, Luo J, Gao G (2015). GSDS 2.0: an upgraded gene feature visualization server. Bioinformatics..

[70] Liu WXY, Ma J, Luo X, Nie P, Zuo Z, Lahrmann U, Zhao Q, Zheng Y, Zhao Y, Xue Y, Ren J (2015). IBS: an illustrator for the presentation and visualization of biological sequences. Bioinformatics..

[71] Li W, Cowley A, Uludag M, Gur T, McWilliam H, Squizzato S, Park YM, Buso N, Lopez R (2015). The EMBL-EBI bioinformatics web and programmatic tools framework. Nucleic Acids Res.

[72] Kumar G, Arya P, Gupta K, Randhawa V, Acharya V, Singh AK (2016). Comparative phylogenetic analysis and transcriptional profiling of MADS-box gene family identified *DAM* and *FLC-like* genes in apple (*Malusx domestica*). Sci Rep.

[73] Verma D, Lakhanpal N, Singh K (2019). Genome-wide identification and characterization of abiotic-stress responsive *SOD* (*superoxide dismutase*) gene family in *Brassica juncea* and *B. rapa*. BMC Genomics.

[74] Lee SC, LM, Kim JA, Lee SI, Kim JS, Jin M, Kwon SJ, Mun JH, Kim YK, Kim HU, Hur Y, Park BS (2008). Transcriptome analysis in *Brassica rapa* under the abiotic stresses using Brassica 24K oligo microarray. Mol Cell.

[75] Xu J, Xing XJ, Tian YS, Peng RH, Xue Y, Zhao W, Yao QH (2015). Transgenic Arabidopsis plants expressing tomato *glutathione S-transferase* showed enhanced resistance to salt and drought stress. PLoS One.

[76] Behrens FH, Schenke D, Hossain R, Ye WZ, Schemmel M, Bergmann T, Hader C, Zhao Y, Ladewig L, Zhu WX (2019). Suppression of abscisic acid biosynthesis at the early infection stage of *Verticillium longisporum* in oilseed rape (*Brassica napus*). Mol Plant Pathol.

[77] Livak KJ, Schmittgen TD (2001). Analysis of relative gene expression data using real-time quantitative PCR and the 2(−Delta Delta C(T)) method. Methods..

